# Targeting peroxiredoxin 1 impairs growth of breast cancer cells and potently sensitises these cells to prooxidant agents

**DOI:** 10.1038/s41416-018-0263-y

**Published:** 2018-10-05

**Authors:** Malgorzata Bajor, Agata O. Zych, Agnieszka Graczyk-Jarzynka, Angelika Muchowicz, Malgorzata Firczuk, Lech Trzeciak, Pawel Gaj, Antoni Domagala, Marta Siernicka, Agnieszka Zagozdzon, Pawel Siedlecki, Monika Kniotek, Patrick C. O’Leary, Jakub Golab, Radoslaw Zagozdzon

**Affiliations:** 1 Department of Immunology, Center of Biostructure Research, Medical University of Warsaw, Warsaw, Poland; 2 Department of Clinical Immunology, Transplantation Institute, Medical University of Warsaw, Warsaw, Poland; 3 Postgraduate School of Molecular Medicine, Medical University of Warsaw, Warsaw, Poland; 4 Department of Medical Genetics, Medical University of Warsaw, Warsaw, Poland; 5 Center for Advanced Materials and Technologies, Warsaw University of Technology, Warsaw, Poland; 6 Laboratory of Human Cancer Genetics, Centre of New Technologies, University of Warsaw, Warsaw, Poland; 7 Department of Bioinformatics, Institute of Biochemistry and Biophysics, Polish Academy of Sciences, Warsaw, Poland; 8 Department of Systems Biology, Institute of Experimental Plant Biology and Biotechnology, University of Warsaw, Warsaw, Poland; 9 Helen Diller Family Comprehensive Cancer Center, University of California San Francisco, San Francisco, CA USA; 10 Centre for Preclinical Research and Technology, Medical University of Warsaw, Warsaw, Poland

**Keywords:** Breast cancer, Target identification

## Abstract

**Background:**

Our previous work has shown peroxiredoxin-1 (PRDX1), one of major antioxidant enzymes, to be a biomarker in human breast cancer. Hereby, we further investigate the role of PRDX1, compared to its close homolog PRDX2, in mammary malignant cells.

**Methods:**

CRISPR/Cas9- or RNAi-based methods were used for genetic targeting PRDX1/2. Cell growth was assessed by crystal violet, EdU incorporation or colony formation assays. In vivo growth was assessed by a xenotransplantation model. Adenanthin was used to inhibit the thioredoxin-dependent antioxidant defense system. The prooxidant agents used were hydrogen peroxide, glucose oxidase and sodium L-ascorbate. A PY1 probe or HyPer-3 biosensor were used to detect hydrogen peroxide content in samples.

**Results:**

PRDX1 downregulation significantly impaired the growth rate of MCF-7 and ZR-75-1 breast cancer cells. Likewise, xenotransplanted PRDX1*-*deficient MCF-7 cells presented a retarded tumour growth. Furthermore, genetic targeting of PRDX1 or adenanthin, but not PRDX2, potently sensitised all six cancer cell lines studied, but not the non-cancerous cells, to glucose oxidase and ascorbate.

**Conclusions:**

Our study pinpoints the dominant role for PRDX1 in management of exogeneous oxidative stress by breast cancer cells and substantiates further exploration of PRDX1 as a target in this disease, especially when combined with prooxidant agents.

## Introduction

Persistently exaggerated oxidative stress in cancers,^1^ including breast cancer, is most often associated with an aggressive phenotype and contributes to changes in signalling pathways, consequently reprograming the cells to adapt to new unfavourable conditions.^2^ Indeed, in order to survive under elevated oxidative stress cancer cells tend to upregulate their antioxidant defenses^3^ and this phenomenon can potentially be targeted for therapeutic applications.^4^ This subject becomes even more attractive with rising hopes for using CRISPR/Cas9-based approaches for genome-specific targeting in cancer.^5^ Given the functional overlaps in antioxidant systems and for the sake of precision therapies, it is necessary to identify the level of dependence of cancer cells on each of the antioxidant molecules.

PRDXs are a group of highly conserved proteins, acting as thiol-dependent scavengers of H_2_O_2_, but also as chaperones or DAMPs (danger-associated molecular patterns).^6^ The human genome encodes six forms of PRDXs (PRDX1-6). Out of these, the subcellular localisation of PRDX1 and its close homolog PRDX2 in the cytosol suggests that they would act as sensors for changes in extracellular concentrations of H_2_O_2_ after this compound enters the cell through the cell membrane. Several studies, including recent publications from our team,^7,8^ have analysed whether PRDXs can be regarded biomarkers in cancers or targets for anticancer therapies. For instance, the previous study from our team suggested peroxiredoxin 1 (PRDX1), and also PRDX2, as druggable targets in Burkitt lymphoma.^8^ Additionally, we have described PRDX1 to be a biomarker in breast cancer.^9^ This led us to a question whether targeting PRDX1 and/or PRDX2 could be a successful therapeutic strategy in this disease. Our current study indicates the superior role for PRDX1, but not PRDX2, in curtailing oxidative stress by breast cancer cells and suggests that this molecule could be further examined as a potential therapeutic target in this disease.

## Methods

### Analysis of human clinical data

The raw HTSeq counts were obtained for the breast cancer paired samples (tumour vs. normal) from the TCGA Research Network.^10^ The differential gene expression analysis was done in the paired sample (*n* = 108) setting of linear regression model for the whole transcriptome (20,063 genes) in edgeR.^11,12^

### Cell lines

Oestrogen receptor(ER)-positive (MCF-7, ZR-75-1 and T47D), triple-negative (MDA-MB-231, HCC 1806), and ER-negative/HER2-positive (SK-BR-3) human breast carcinoma cell lines and MCF-10A, a non-malignant immortalised mammary cell line, were purchased from European Collection of Cell Cultures (Wiltshire, UK). HMEC, primary human mammary epithelial cells were purchased from Life Technologies (Carlsbad, CA). HEK-293FT cells were purchased from Invitrogen (Carlsbad, CA) as a part of BLOCK-iT Lentiviral RNAi Expression System. HEK293-T cells were obtained from DSMZ (Germany), as reported previously.^7^ See Supplementary Methods for culturing conditions.

### Generation of individual PRDX1- or PRDX2- knockout cell lines by CRISPR/Cas-mediated genome editing for in vitro study

The lentiviral vector lentiCRISPR v2 was a gift from Feng Zhang (Addgene plasmid #52961,^13^). sgRNA sequences towards PRDX1 or PRDX2 were designed using E-CRISP website. Sequences with no mismatch targets were chosen for further steps (see Suppl. Table S1). From the selected sgRNAs, one sgRNA sequence was chosen for each gene: PRDX1#14 and PRDX2#16, further on referred to as sgPRDX1 and sgPRDX2, respectively. As controls, either a construct with sgRNA towards GFP gene (not present in human genome) or a non-mammalian targeting control (NTC) were used. Cloning of CRISPR constructs encoding these sgRNAs was done according to the protocol described by Shalem et al.^13^ Next, using the Lipofectamine 2000 Reagent (Invitrogen, Carlsbad, California, USA) human embryonic kidney (HEK)-293FT cells (60–70% confluency) were transfected with the sgRNA-expressing plasmids together with packaging and envelope plasmids (psPAX2 and pMD2.G, respectively) according to the manufacturer’s protocol. The next day medium was refreshed, and cells were cultured for next 24 h. Then, lentivirus-containing supernatant was harvested, filtered through 0.45 µm pore size filter and added to 50% confluent MCF-7 target cells. After an additional 24 h, the medium in the MCF-7 cell culture was replaced with fresh aliquots of the respective filtered viral supernatant. After transduction, cells were grown in presence of selective antibiotic, puromycin, at 2 μg/ml for five days. Then, genetically-engineered clones of MCF-7 cells were obtained by seeding the cells at a calculated density 0.5 cells per well onto 96-well plates, and then western blotting quantification of the expanded cell cultures was performed. The exact genetic alteration at the sgRNA-targeting locus of PRDX1 and 2 was identified by mRNA sequencing as described in the Supplementary Results.

### RNA sequencing

Sequencing was performed using BigDye Terminator v3.1 Cycle Sequencing Kit (Applied Biosystems) on a 3500xL Genetic Analyzer (Applied Biosystems). Detailed information is provided in Supplementary Methods.

### EdU assay

The rate of cell proliferation was assessed using EdU assay (C10420, Life Technologies). Cells were incubated with 50 μM 5-ethynyl-2′-deoxyuridine (EdU) for 2 h and then harvested, fixed, permeabilized, and labeled according to Click-iT® EdU Alexa Fluor® 488 Imaging Kit protocol. The percentage of EdU-positive cells was evaluated by flow cytometry using Accuri C6 device (BD Biosciences).

### Cell cycle

Cell cycle analysis was performed as described previously^8^.

### Generation of MCF-7 sgNTC and sgPRDX1-pool cells for in vivo study

To construct pLenti7.3/V5 TOPO-RedLuc vector, the red luciferase gene was amplified by PCR from a plasmid pMCS-Red Firefly Luc (Thermo Fisher) using sense primer 5′ ACCATGGAAAATATGGAAAACGACGAG -3′ and antisense primer 5′- TCACATCTTGGCCACGGGTTT -3′. The PCR product was gel-purified and cloned into pLenti7.3/V5 TOPO (Thermo Fisher). For details of lentiviral transduction protocol see Supplementary Methods.

### Mice

6–8-week-old BALB/c-Nude (CAnN.Cg-Foxn1nu/Crl) females were obtained from the Charles River Laboratories (USA).

### Tumour treatment and monitoring

Slow-release pellets containing 0.36 mg of 17β-estradiol (Innovative Research of America, USA), were implanted subcutaneously three days before tumour cells inoculation. At the day 0, MCF-7-sgNTC-pool2 and MCF-7-sgPRDX1-pool2 cells were harvested, washed, and resuspended in PBS. Subsequently, 3 × 10^6^ cells were inoculated into the second, left mammary fat pad of experimental mice in 50% Matrigel (BD, USA). Tumour growth was monitored one time per week with calipers. Once the first tumours reached the size of humane endpoint (i.e., 15 mm diameter), all mice were ethically sacrificed.

### Generation of HyPer-3-expressing MCF-7 cell lines

The pC1-HyPer-3 was a gift from Vsevolod Belousov (Addgene plasmid #42131).^14^ Next, HyPer-3 coding fragment was cloned into a pHIV-SFFV-mRFP-WPRE plasmid-based vector behind the SFFV promoter by replacing of mRFP gene. Human embryonic kidney (HEK)-293T cells (60–70% confluency) were transfected with the pHIV-SFFV-HyPer-3-WPRE vector together with packaging and envelope plasmids (psPAX2 and pMD2.G, respectively) using polyethylenimine (PEI) transfection reagent. The lentiviral transduction of MCF-7 target cells was performed as described above. Then, the HyPer-3-positive clones of MCF-7 cells were obtained by seeding the cells at a calculated density 0.5 cells per well onto 96-well plates. The expanded MCF-7-HyPer-3 cell cultures were then modified in parallel using sgNTC (controls) or sgRNA sequence towards PRDX1 by CRISPR/Cas9-based method and further used for imaging studies. In consequence, two engineered cell pools were generated: sgNTC-pool3 and sgPRDX1-pool3, respectively.

### Intracellular H_2_O_2_ imaging in MCF-7-HyPer-3 cells

Long-term live fluorescence imaging was performed using Lumascope 720 device (Etaluma Inc, Carlsbad, CA) under controlled atmosphere (5% CO_2_) and temperature (37 °C) and a ×20 magnification objective. One day before imaging, cells were seeded onto 96-well, poly-D-lysine coated plates with glass clear bottom and black well walls (Perkin Elmer). To reduce background fluorescence, cells were seeded in FluoroBrite DMEM (Life Technologies) medium supplemented with 10% FBS, 1% Pen/Strep, and 2 mM L-glutamine, at a density of 1.2 × 10^5^ per well. The next day, cells were treated with 100 µM H_2_O_2_ dissolved in medium. Consecutive images were acquired at defined time points for 1.5 h using the green fluorescence channel. Image processing was performed with Lumaquant software (Etaluma).

### Chemical reagents

Glucose oxidase (GOx), sodium L-ascorbate (L-ASC), and catalase from bovine liver were obtained from Sigma Aldrich (St Louis, MO, USA), the reagents were dissolved in sterile distilled water. Hydrogen peroxide (H_2_O_2_) was dissolved in appropriate medium. Adenanthin (ADNT) was purchased from Faces Biochemical Co. (Wuhan, China) and dissolved in DMSO.

### Stable shRNA-mediated knockdown of PRDX1 and PRDX2

Lentivirus-mediated knockdown of either PRDX1 or PRDX2 in breast cancer cell lines (ZR-75-1, T47D, MDA-MB-231, HCC 1806, SK-BR-3) and non-malignant MCF-10A cell line was performed as described previously.^9^ shRNA modified cells with high expression of GFP were sorted with FACSAria III cell sorter (BD Biosciences, La Jolla, CA, USA).

### In vitro combinations with prooxidant agents

Cells were seeded onto 96-well plates at a density of 1.2–1.5 × 10^4^ per well for 24 h. To assess cytotoxicity of the prooxidant compounds cells were treated with ADNT alone or in combination with either GOx or L-ASC for 48 h. Next, supernatants were discarded and the cells were stained by crystal violet. Drug combination studies and their synergy quantification were calculated using the Chou–Talalay method by CompuSyn software. The observed values in all treatment groups were normalised to untreated control. According to obtained effects of individual drug treatment and drugs in combination the resulting combination index (CI) for additive effect (CI = 1), synergism (CI < 1), and antagonism (CI > 1) were calculated.^15^ Experiments were performed at least in triplicates.

### Site-directed mutagenesis of PRDX1 cDNA

The cysteine-to-alanine mutations were introduced to pLenti6/V5-DEST-PRDX1 using QuikChange XL Site-Directed Mutagenesis Kit (Agilent Technologies, USA), as described previously.^8^ Next, cDNAs encoding the mutated forms of PRDX1 were cloned into pLenti7.3/V5-TOPO® vector (Thermo Fisher) using sense primer 5′- CTAATGTCTTCAGGAAATGCTAAAATTGGG-3′ and antisense primer 5′-CTCACTTCTGCTTGGAGAAATATTCTTTGC-3′ according to manufacturer’s instructions. Mutations were confirmed by DNA sequencing. Following lentiviral transduction, the GFP-positive cells were sorted with FACSAria III cell sorter (BD Biosciences, La Jolla, CA, USA).

### DFX treatment

A total of 1.2 × 10^4^ cells were seeded onto 96-well plate and allowed to adhere overnight. Next, 0.4 mM L-ASC or 0.5 mU/ml GOx and 31.25 µM deferoxamine (DFX) alone or in combination were added for 48 h. Cell morphology was assessed microscopically (inverted microscope, ×20 magnification, Nikon), and cell viability was determined by crystal violet staining.

### Statistical analysis

All experiments were performed independently at least three times, unless otherwise stated. Statistical analysis was performed with GraphPad Prism. Results are shown as mean ± standard error of the means (S.E.M.) for repeated experiments, unless otherwise stated. The differences between groups were analysed using Student’s *t*-test (only two groups), one-way analysis of variance (more than two groups compared), or two-way analysis of variance (when two independent variables compared). Significant one-way ANOVA results were followed up with a Tukey’s honestly significant difference (HSD) post hoc test. *P* values of less than 0.05 were considered significant.

### Other methods used

Please refer to the Supplementary Methods for additional description of methodology.

## Results

### Expression of both PRDX1 and PRDX2 is highly upregulated in breast cancers

**Fig. 1:**
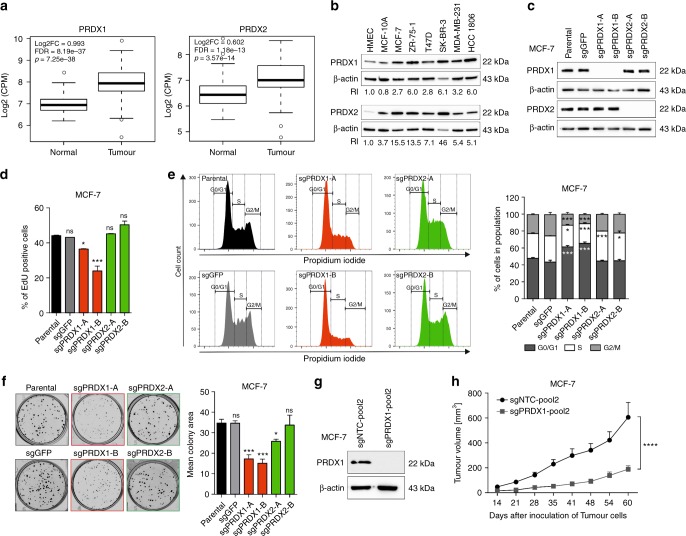
Characterisation of PRDX1 and PRDX2 knockout in MCF-7 breast cancer cell line and in in vivo xenotransplantation model. **a** Analysis of the expression of PRDX1 (left panel) and PRDX2 (right panel) mRNA in normal and breast cancer tissues available in the analysed TCGA dataset (*n* = 108 pairs), regardless of the ethnicity of the patient. **b** Representative western blotting results showing the protein presence of PRDX1 and PRDX2 in HMEC, MCF-10A, MCF-7, ZR-75-1, T47D, SK-BR-3, MDA-MB-231, and HCC1806 cell lines. β-actin was used as a loading control. Bands were quantified by densitometry, RI was calculated as the quotient of the densitometry signal for PRDX1 (or PRDX2) band and that for β-actin and then normalised to that of the HMEC. Averaged RI value from two independent experiments was shown. **c** Western blotting shows efficient depletion of PRDX1 or PRDX2 proteins in the MCF-7 single-cell-derived clonal lines compared to parental and sgGFP controls. β-actin was used as a loading control. **d** Detection of DNA synthesis using EdU incorporation assay in PRDX1-knockout MCF-7 cells (red bars) compared to controls (black and grey bars) and PRDX2-knockout cells (green bars). *p < 0.05, ***p < 0.001. **e** Cell cycle analysis evaluated by the propidium iodide flow cytometry-based assay. Representative cell cycle profiles showing the distribution of cells in the different phases of the cell cycle for PRDX1-knockout MCF-7 cells compared to controls and PRDX2-knockout cells are presented (left panel). Summary of the percentage of cells in each phase of the cycle (right panel). Data shown are cumulative results from two independent experiments performed in triplicates, *p < 0.05, ***p < 0.001. **f** Representative images for the colony formation in MCF-7 cells (left panel) and a quantitative analysis of colony formation assay in PRDX1-knockout MCF-7 cells. Data shown are cumulative results from three independent experiments. *p < 0.05, ***p < 0.001. **g** Western blotting shows efficient depletion of PRDX1 protein in MCF-7-sgPRDX1-pool2 cells as compared to sgNTC-pool2 control. β-actin was used as a loading control. **h** Plots of mean tumour volumes in mice (initial *n* = 10 per group) inoculated with control (sgNTC-pool2) and sgPRDX1-pool2 of MCF-7 cells. Points are means and bars are SD. Statistical analysis was performed with two-way Anova test (****p < 0.0001)

Previous reports suggested that peroxiredoxins can be significantly upregulated in mammary malignancies.^16^ Hereby, we have analysed the publicly accessible data derived of the TCGA Research Network. As shown in Fig. 1a, based on 108 cases analysed we observed that transcripts for both PRDX1 and PRDX2 are markedly elevated in malignant tissues when compared to the matched healthy specimens. Likewise, when a range of breast cancer cell lines were analysed by western blotting (Fig. 1b), we noticed that PRDX1 and PRDX2 protein content is highly upregulated in breast cancer cells, as compared to primary human mammary epithelial cells HMEC and non-malignant, mammary tissue-derived MCF-10A cell line. We seek to validate the dependence of mammary tumour cell survival on an increased expression of PRDX1 or PRDX2 within the current study.

### PRDX1, but not PRDX2 knockout, reduces growth rate of MCF-7 breast cancer cells

To downregulate PRDX1 or PRDX2, we used the genome-targeted knockout in MCF-7 cells and the RNAi-based knockdown in other breast cancer cell lines (described below). In the knockout approach, we have utilised a technique based on clustered regularly interspaced short palindromic repeats RNA-guided Cas9 nucleases (CRISPR/Cas9; see Suppl. Table S1 for all sgRNA sequences). For the sake of clarity, the abbreviated names for all CRISPR/Cas9-modified MCF-7-derived cell lines are presented in Supplementary Table S2.

Out of two sgRNAs-targeting PRDX1, we observed a significant reduction of PRDX1 protein content in case of sgRNA #14 (Suppl. Fig. S1A), which was used subsequently. For functional validation, we subjected the MCF-7 sgGFP-pool1 and sgPRDX1-pool1 cells to Cs-137-irradiation, a common model for inducing excessive ROS production,^17^ and which PRDX1 is suggested to protect the cells against.^18^ Indeed, we observed a significant sensitisation of sgPRDX1-pool1 cells to the toxicity of irradiation as compared to the controls (Suppl. Fig. S1B), which validated the applicability of CRISPR/Cas9 technology for functional studies with PRDX1. In parallel, we assessed the effects of four sgRNAs-targeting PRDX2 in the pooled MCF-7 cells (Suppl. Fig. S1C) and we have chosen sgPRDX2 (#16) for further studies.

Then, two pairs of PRDX1/2-deficient clones (referred to as clones sgPRDX1-A and -B for PRDX1 knockout, and sgPRDX2-A and -B for PRDX2 knockout) were selected for further analyses. As depicted in Fig. 1c, in western blotting the selected clones showed a complete absence of either PRDX1 or PRDX2 expression as compared to the parental (unedited) and sgGFP controls. To identify specific genomic changes for each selected clone, we sequenced the PRDX1 or PRDX2 mRNAs and the graphical representation of the results is shown in Supplementary Fig. S1D, E (see also Supplementary Results).

During phenotypic characterisation of the selected clones, we have observed a significantly decreased EdU incorporation into both MCF-7 sgPRDX1-A and -B clones, whereas the percentage of EdU-positive cells in MCF-7 controls (parental and sgGFP) and both sgPRDX2 clones exhibited no apparent differences (Fig. 1d). Accordingly, in MCF-7 cells with PRDX1 knockout, but not with PRDX2 knockout, we observed a statistically significant G0/G1 cell cycle arrest (Fig. 1e). Likewise, colony formation assays revealed that MCF-7 sgPRDX1-deficient, but not sgPRDX2-deficient clones, exhibited pronounced growth impairment, as compared to controls (Fig. 1f).

### PRDX1 knockout inhibits in vivo growth of mammary tumours derived from MCF-7 cells

As in vivo growth can be characterised with different oxidative stress conditions than in vitro culture,^19^ we studied the effects of PRDX1 knockout on the growth of MCF-7 cells xenotransplanted to nude mice. To avoid the effects of clonal selection, we have utilised newly generated pools (referred to as ‘pool2’) of MCF-7 cells transduced with either control sgNTC or sgPRDX1-encoding vectors (Fig. 1g and Suppl. Fig. S2A). When implanted into mammary fat pad of the nude mice, MCF-7 sgPRDX1-pool2 cells showed a significant tumour growth retardation in comparison with control (Fig. 1h and Suppl. Fig. S2B), which corroborates the growth-inhibitory effects of genetic targeting PRDX1 also in vivo.

### Knockout of PRDX1 disturbs intracellular metabolism of exogeneous hydrogen peroxide in MCF-7 cells

**Fig. 2:**
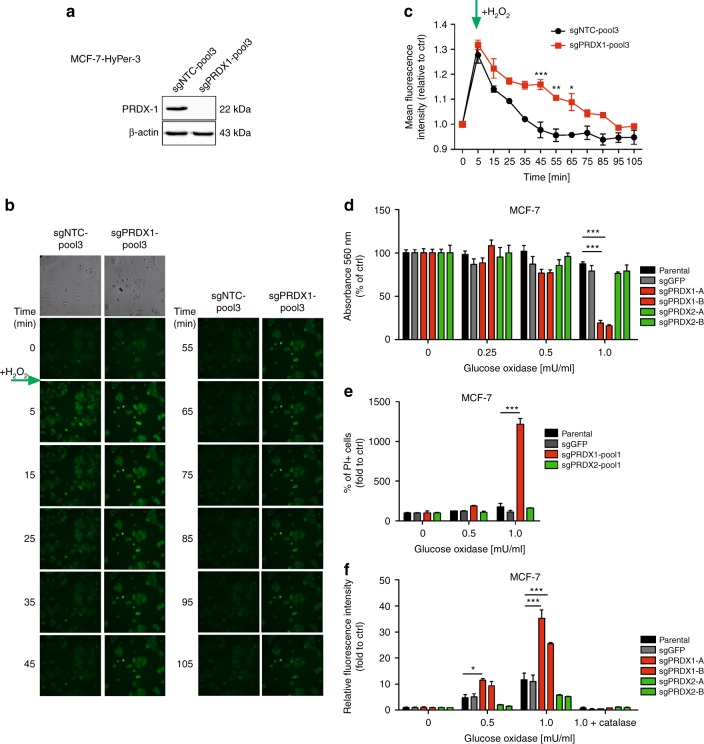
Assessment of cellular responses to H_2_O_2_ using MCF-7 cells. **a** Western blotting shows efficient depletion of PRDX1 protein in the MCF-7-HyPer-3 cells. **b** Fluorescence of HyPer-3 (green) in live sgNTC-pool3 and sgPRDX1-pool3 MCF-7 cells after adding exogenous H_2_O_2_ at final concentration of 100 µM at the indicated time points. **c** Fluorescence intensities of HyPer-3 in the sgNTC-pool3 and sgPRDX1-pool3 MCF-7 cells untreated and incubated with 100 µM H_2_O_2_. Green arrow indicates adding of H_2_O_2_. Experiment was performed twice. **d** Concentration-dependent cytotoxicity of H_2_O_2_ produced by GOx in CRISPR/Cas9-engineered MCF-7 cells. Cells were treated with a range of GOx concentrations (0.25; 0.5; 1 mU/ml) for 24 h. Control cells were cultured without any reagent. At the end of treatment, the crystal violet staining (or MTT test, see Suppl. Fig. S3A) was performed and reported as percent growth relative to control. **e** Determination of cell death by propidium iodide staining followed by flow cytometry analysis. Cells were treated with a range of GOx concentrations (0.5; 1 mU/ml) for 24 h. Control cells were cultured without any reagent. Experiments were performed in duplicates and repeated three times, ***p < 0.001. **f** Relative fluorescence intensity (log_2_ values) of PY1 probe after reaction with H_2_O_2_ produced by GOx in sgPRDX1 and sgPRDX2 MCF-7 cells compared to controls. Cells treated with 1 mU/ml of GOx were preincubated with catalase (100 μg/ml) for 30 min. After 24 h of GOx treatment 10 μM PY1 dye was added to the medium for 30 min at 37 °C and the read was taken using EnVision reader at the excitation wavelength 514 nm and emission wavelength 550 nm. Each sample was at least triplicated, and data were obtained from three independent experiments. Statistical analysis was performed with one-way ANOVA followed by Tukey’s honestly significant difference (HSD) post hoc test when significance was detected (**p < 0.01, ***p < 0.001)

To study an intracellular dynamics of coping with exogeneous oxidative insult by MCF-7 cells devoid of PRDX1, we have engineered MCF-7 parental cells to express a H_2_O_2_-specific sensor—HyPer-3.^14^ Then, CRISPR/Cas9-mediated knockout of PRDX1 was carried out, which produced MCF-7 sgPRDX1-pool3 cells and, parallelly, the sgNTC-pool3 controls were generated (Fig. 2a). The newly engineered cells were subjected to live-cell fluorescent imaging after adding exogenous H_2_O_2_ at a final concentration of 100 µM to cell cultures. As depicted in Fig. 2b and C, MCF-7-sgPRDX1-pool3 cells were characterised by prolonged retention of increased HyPer-3 fluorescence as compared to the controls. That corroborates the metabolic dependence of MCF-7 cells on PRDX1 regarding removal of exogenous H_2_O_2_.

### Knockout of PRDX1, but not PRDX2, dramatically reduces survival of MCF-7 cells under exogenous oxidative stress conditions

Since PRDX1 and PRDX2 are cytosolic sensors and scavengers of peroxides, to study the effects of a sustained exposure to elevated H_2_O_2_ levels, cells were treated with increasing concentrations (0–1 mU/ml) of GOx followed by viability and cell death measurement using crystal violet staining and a propidium iodide flow cytometry-based assay, respectively. As shown in Fig. 2d, e, exposure of MCF-7 sgPRDX1-A/-B clones and MCF-7 sgPRDX1-pool1 to GOx significantly decreases cells’ viability and cytotoxicity, respectively, while the controls and sgPRDX2-A/-B clones, as well as MCF-7 sgPRDX2-pool1 showed no impairment in survival. Importantly, this effect was completely abrogated by an addition of catalase to the cell culture indicating that the effect was solely dependent on extracellular production of H_2_O_2_ (Suppl. Fig. S3A). To study the potential changes in H_2_O_2_ turnover by the PRDX1/2-deficient cells, we assessed the overall content of H_2_O_2_ in all cell cultures using the fluorescent boronate PY1 probe.^20,21^ We observed that the PY1 fluorescence was markedly increased upon GOx treatment in MCF-7 sgPRDX1-A/-B cells as compared to that in the control counterparts and MCF-7 sgPRDX2-A/-B cells (Fig. 2f).

### Knockout of PRDX1 changes functional status of remaining 2-Cys PRDXs in MCF-7 cells

Elevated hydrogen peroxide concentrations in cells trigger PRDX1-4 hyperoxidation, which hampers their antioxidant activity.^22^ Notably, PRDX1 was published to become preferentially hyperoxidated in some experimental models and acting as a “shield” for other PRDXs.^23^ To gain a deeper insight in the current work into the biochemical effects of PRDX1 knockout in MCF-7 cells, we have evaluated the hyperoxidation status of 2-Cys PRDXs in the steady state and in response to glucose oxidase (GOx) treatment. As expected, in parental MCF-7 controls we observed a gradual increase in hyperoxidation levels of PRDX1-4 correlating with the GOx concentrations used (Suppl. Fig. S3B). In turn, in case of MCF-7-sgPRDX1-B cells the hyperoxidation levels of PRDX2-4 were initially markedly higher than in controls, and, for PRDX2 and 4, further increased after incubation with GOx. This corroborates the notion of PRDX1 acting as a “shield” for other 2-Cys PRDXs, and indicates one of the potential explanatory mechanisms for the particular role for PRDX1 in protection of breast cancer cells against oxidative stress.

Additionally, to get an insight into the effects of PRDX1 knockdown on the balance between dimeric and monomeric forms of other PRDXs, we followed previous publication on this topic^24^ and assessed the dimerisation status of PRDX3 after H_2_O_2_ treatment in MCF-7 cells. As shown in Supplementary Figure S3C, in both sgGFP controls and PRDX1-deficient cells alike there was an abrupt shift from dimeric into monomeric form of PRDX3 after 5-minute incubation with 100 μM H_2_O_2_. However, in the control cells there was a visible recovery of PRDX3 dimer after 30 min from the addition of H_2_O_2_ to the cell culture, while in PRDX1-deficient cells there was a marked progression of monomerisation of PRDX3. This, along with the results presented in Fig. 2b, c, suggests that the impairment of catabolism of exogenous H_2_O_2_ in PRDX-deficient cells translates into an enhancement of dysfunction of remaining PRDXs under oxidative stress conditions, as exemplified by PRDX3 hereby. This again indicates that inactivity of other PRDXs, e.g. PRDX3, can be responsible for the effects of increased sensitivity to prooxidants observed in PRDX1-deficient cells.

### RNAi-based downregulation of PRDX1, but not PRDX2, reduces the growth rate and response to oxidative stress of ZR-75-1 breast cancer cells

**Fig. 3:**
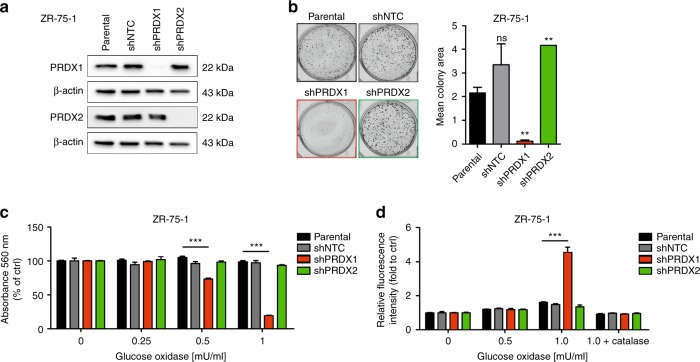
Characterisation of PRDX1 and PRDX2-knockdown ZR-75-1 breast cancer cell line. **a** Western blotting shows downregulation of PRDX1 or PRDX2 proteins in ZR-75-1 cell line derivatives compared to parental and shNTC controls. β-actin was used as a loading control. **b** Representative images for the colony formation in ZR-75-1 cells (left panel) and a quantitative analysis of colony formation assay (right panel) shows a significant decrease of colony number in cells carrying shPRDX1, in contrast to controls and PRDX2-knockdown cells. Data shown are cumulative results from three independent experiments. **p < 0.01. **c** Concentration-dependent cytotoxicity of GOx in ZR-75-1 cells. Cells were treated with GOx (0.25; 0.5; 1 mU/ml) for 24 h. Control cells were cultured without any reagent. At the end of treatment, the crystal violet staining (or MTT test, see Suppl. Fig. S3D) was performed and reported as percent growth relative to control. **d** Relative fluorescence intensity (log_2_ values) of PY1 probe after reaction with H_2_O_2_ produced by GOx in shPRDX1 and shPRDX2 ZR-75-1 cells compared to controls was assessed as described in Fig. 2e

For the validation purposes, we have used the previously described^9^ shRNA-encoding vectors to knockdown either PRDX1 or PRDX2 in another model cell line for oestrogen receptor positive breast cancer, ZR-75-1 (Fig. 3a). Hereby, similarly to MCF-7, we observed a significant decrease in the growth rate of ZR-75-1 breast cancer cells harboring the shRNA against PRDX1 (ZR-75-1 shPRDX1), but not PRDX2 (ZR-75-1 shPRDX2), as compared to the controls (ZR-75-1 parental and shNTC) (Fig. 3b). We have also confirmed a remarkable sensitisation of the ZR-75-1 shPRDX1, but not shPRDX2, cells to GOx-induced oxidative stress as compared to the controls (Fig. 3c and Suppl. Fig. S3D). Again, this effect was abrogated by catalase (Suppl. Fig. S3D) and correlated with a deep impairment in H_2_O_2_ metabolism by ZR-75-1 shPRDX1 cells, as evaluated by the PY1 sensor probe (Fig. 3d). Taken together, these findings imply again that mainly PRDX1, but not PRDX2, plays a substantial role in exogenous H_2_O_2_ metabolism in breast cancer cells and, hence, is protecting these cells against toxicities of the H_2_O_2_-stimulated oxidative stress.

### Downregulation of PRDX1 and PRDX2 in breast cancer cells are differently reflected by Akt phosphorylation

As both PRDX1^9^ and 2^25^ have been previously shown to regulate the prosurvival PI3K/Akt-mediated signalling in a cell type-dependent manner, we assessed the Ser_473_Akt phosphorylation status in MCF-7 cells devoid of either PRDX1 or PRDX2. As shown in Supplementary Fig. S3E, there was a stark difference between both cell types—while knockout of PRDX1 did not change the phosphorylation status of Akt over controls in steady state, the PRDX2 knockout induced a marked elevation of Ser_473_Akt phosphorylation. Moreover, when cells were subjected to exogenous oxidative stress by incubation with GOx, in PRDX1 knockout cells there was a moderate level of increase of Ser_473_Akt phosphorylation status in high concentrations (0.5–2 U/ml) of GOx, when compared to control cell lines (Suppl. Fig. S3F, left panels), which corresponds with our previously published observation in ZR-75-1 cells.^9^ Conspicuously, in PRDX2 knockout cells the Ser_473_Akt remained constantly at high levels (Suppl. Fig. S3F, right panels). Our data suggest that the difference in regulation of Akt-mediated prosurvival signalling pathway may be partially responsible for differential response of PRDX1- versus PRDX2-deficient cells to oxidative stress.

### Downregulation of PRDX1, but not PRDX2, potently sensitises breast cancer cells to ascorbate-induced toxicity

Our observations of the dependence of breast cancer cell survival on PRDX1 under the oxidative stress conditions, prompted us to combine PRDX1-targeting with the clinically applicable prooxidant agent, sodium L-ascorbate (L-ASC).^26^ When used in concentrations higher than physiological, L-ASC is known to generate excessive quantities of hydrogen peroxide.^27^

**Fig. 4:**
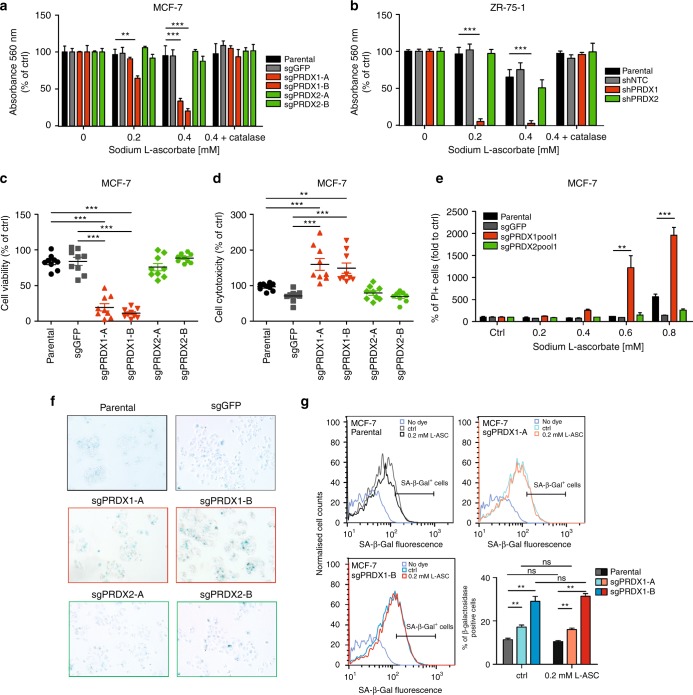
Knockout of PRDX1, but not PRDX2, sensitises breast cancer cells to prooxidant agents. MCF-7 (**a**) and ZR-75-1 (**b**) cells were treated with increasing concentration of sodium ascorbate (0.2, 0.4 mM) for 24 h. Cells treated with 0.4 mM of L-ASC were preincubated with catalase (100 μg/ml) for 30 min. **b**, **c** Effects of 0.4 mM L-ASC treatment for 24 h on MCF-7 control, sgPRDX1 and 2 cells viability (**c**) and cytotoxicity (**d**). Fluorescence was measured at 400_Ex_/505_Em_ (viability) and 485_Ex_/520_Em_ (cytotoxicity) using EnVision reader. Each sample was at least triplicated, and data were obtained from three independent experiments. **e** Determination of cell death by propidium iodide staining followed by flow cytometry analysis. Cells were treated with a range of L-ASC concentrations (0.2–0.8 mM) for 24 h. Control cells were cultured without any reagent. Experiments were performed in duplicates and repeated three times, **p < 0.01, ***p < 0.001. **f** Expression of the senescence marker SA–β-Gal in parental and CRISPR/Cas9-engineered MCF-7 cells was detected by X-Gal staining at pH 6. Microphotographs were taken at 10 magnification (inverted microscope, Nikon). **g** Representative flow cytometry histograms of SA-β-Gal activity and bar graphs showing flow cytometry analysis of SA-β-Gal activity for MCF-7 parental, sgPRDX1-A and sgPRDX1-B cells control (untreated) and after treatment with 0.2 mM L-ASC for 24 h. Experiments were performed in triplicates and repeated twice.**p < 0.01

To this end, genetically modified MCF-7 and ZR-75-1 breast cancer cells were incubated with increasing concentrations of L-ASC (0.2 mM and 0.4 mM) for 24 h followed by crystal violet staining. Interestingly, PRDX1-, but not PRDX2-knockout, dramatically enhanced MCF-7 cell sensitivity to L-ASC (Fig. 4a). The same phenomenon was observed in ZR-75-1 cell line expressing shPRDX1, but not shPRDX2 or the controls (parental and shNTC) (Fig. 4b). The L-ASC-induced growth inhibition was abrogated when cells were pretreated with catalase in the medium. To confirm that the observed effects involve cytotoxicity, the live-cell protease activity (with GF-AFC substrate, Fig. 4c) and dead-cell protease activity assays (with bis-AAF-R110 substrate, Fig. 4d)^28^, as well as PI staining followed by flow cytometry analysis (Fig. 4e) were used.

Additionally, as PRDX1 was previously shown to protect the cells from ROS-induced senescence,^29^ we have assessed senescence associated β-galactosidase (SA-β-gal) activity in PRDX1- versus PRDX2-knockout MCF-7 cells after incubation with X-Gal substrate. As shown in Fig. 4f, there was a tendency towards higher SA-β-Gal activity in PRDX1-deficient MCF-7 cells. Therefore, we have applied a flow cytometry-based method to validate and quantify the SA-β-Gal activity in steady state, and also the effects of L-ASC treatment on this parameter. As shown in Fig. 4g, both clones lacking PRDX1 have presented significant increase in SA-β-Gal activity over parental controls. However, we did not observe any significant changes following incubation of the cells with L-ASC for 24 h. It suggests that although steady-state growth retardation might be senescence associated in our experimental model, an increase in L-ASC toxicity towards the PRDX1-deficient cells should not be considered senescence-mediated.

To study the role for generation of H_2_O_2_ by L-ASC in more details, we have utilised the fact that L-ASC can generate H_2_O_2_ only in presence of iron ions,^27^ while these are not necessary for H_2_O_2_ generation by GOx. Therefore, we have used DFX, a known iron chelator, to combine with L-ASC or GOx treatment of MCF-7 cells with PRDX1 knockout. As expected, DFX, although somewhat growth-inhibitory on its own, alleviated the effects of L-ASC (Suppl. Figs. S4A, B) on MCF-7-sgPRDX1 cells, but had no significant effects on the toxicity of GOx (Suppl. Fig. S4C).

### PRDX1 downregulation sensitises breast cancer cells, but not the non-transformed mammary cells, to prooxidative agents regardless of the molecular subtype of the cell line

As both MCF-7 and ZR-75-1 cells belong to ER-positive subtype of breast cancer, in order to assess other subtypes of mammary carcinomas, we have carried out PRDX1 knockdown in five additional cell lines: non-malignant MCF-10A,^30^ T47D (ER-positive cancer), MDA-MB-231 (triple-negative cancer), HCC 1806 (triple-negative cancer), and SK-BR-3 (HER2-positive, ER-negative cancer). As shown in Supplementary Figure S5, the cell lines were variably sensitive to prooxidant compounds (GOx and L-ASC). The most resistant were the non-malignant MCF-10A cells, and the knockdown of PRDX1 did not produce any amplification of the effects of the prooxidant compounds in this cell line (Suppl. Fig. S5A–C). Conversely, PRDX1 knockdown significantly sensitised all remaining breast cancer cell lines to prooxidant compounds, although this sensitisation was the least pronounced in T47D cell line (Suppl. Fig. S5D–F). Altogether, these results suggest the universality of the effects of PRDX1 targeting among various molecular types of mammary carcinomas.

Furthermore, to confirm the particular role for PRDX1 versus PRDX2 in management of oxidative stress in breast cancer cells, we have generated PRDX2 knockdown in MDA-MB-231 and SK-BR-3 cells (Suppl. Figs S6A and D, respectively). Similarly to MCF-7 and ZR-75-1 models, suppression of PRDX2 failed to sensitise MDA-MB-231 and SK-BR-3 cell lines to the toxicity of prooxidant compounds (Suppl. Fig. S6).

### Catalytic activity of PRDX1 is necessary to rescue the PRDX1-knockout phenotype in MCF-7 cells

**Fig. 5:**
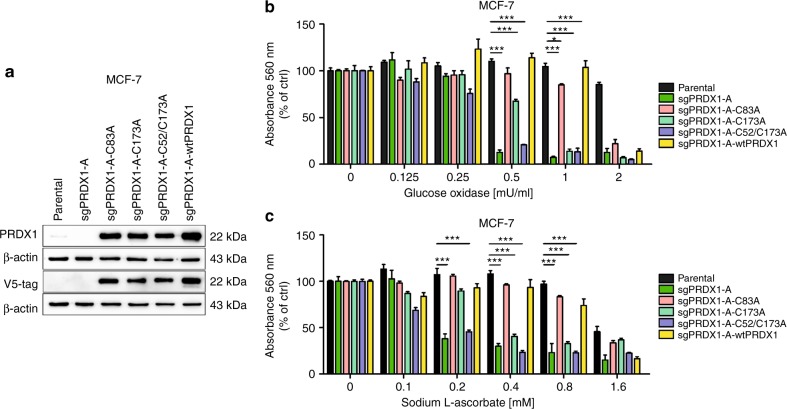
Catalytically inactive PRDX1 variants, in contrast to C83A mutant and wtPRDX1 variant, do not rescue survival of PRDX1-knockout MCF-7 cells. **a** anti-PRDX1 and anti-V5-tag western blotting analysis shows overexpression of PRDX1 mutated protein variants with the following mutations: C83A, C173A, and C52/C173A (lanes 3–5, respectively) or overexpressing wtPRDX1 protein (lane 6) in MCF-7 sgPRDX1-A cells compared to parental (lane 1) and knockout sgPRDX1-A (lane 2) cells. β-actin was used as a loading control. **b**, **c** Cells were treated with increasing concentrations of glucose oxidase (0.125–2 mU) (**b**) or sodium L-ascorbate (0.1–1.6 mM) (**c**) for 24 h. Catalytically inactive PRDX1 variants, in contrast to C83A mutant and wtPRDX1 variant, do not rescue survival of PRDX1-knockout MCF-7 cells. For all cytotoxicity assays, control cells were cultured without any reagent. At the end of treatment, the crystal violet staining was performed and reported as percent growth relative to control. Experiments were performed in triplicates and repeated at least twice. Statistical analysis was performed with one-way ANOVA followed by Tukey’s honestly significant difference (HSD) post hoc test when significance was detected (*p < 0.05, **p < 0.01, ***p < 0.001)

As PRDX1 is a multifunctional molecule, we have assessed whether complementary expression of various functionally impaired forms of PRDX1 can rescue the susceptibility to oxidative stress in PRDX1-knockout MCF-7 cells. To this end, we have used a catalytically active C83A point mutated variant with impaired formation of decameric forms of PRDX1^31^ and two catalytically-impaired mutants: C173A and C52/C173A, and also the wild-type PRDX1 (wtPRDX1), as a positive control for rescue. Each of these forms of PRDX1 was introduced into MCF-7-sgPRDX1-A cells (Fig. 5a). As depicted in Fig. 5b, c, expression of wtPRDX1 or C83A mutant fully rescued the MCF-7-sgPRDX1-A cells from toxic effects of GOx and L-ASC, respectively. Neither C173A nor C52/C173A double mutant was capable of rescuing the PRDX1-knockout cells under such experimental conditions.

### Adenanthin treatment synergistically potentiates prooxidant-induced cytotoxicity in breast cancer cells

Recently, adenanthin (ADNT), a diterpenoid compound isolated from the herb Isodon adenantha,^32^ has been described, also by our group,^9,33,34^ as an inhibitor of PRDX-related antioxidant chain. Hereby, in MCF-7 and ZR-75-1 cell lines we have observed that ADNT disrupted formation of the enzymatically active homodimeric forms of PRDXs, which was accompanied by accumulation of the enzymatically inactive monomers (Suppl. Fig. S7A, B, respectively). The most sensitive PRDXs to ADNT treatment was PRDX1, then PRDX2, and slightly PRDX3, whereas PRDX4 retained their dimeric forms at up to 2 micromolar concentrations of ADNT. To get insight into molecular basis of this phenomenon, we have examined the amino acid sequences of 2-Cys human PRDXs and their possible interaction with ADNT (Suppl. Fig. S7C). Our analysis supports the notion of preference of ADNT binding to PRDX1 and 2 as compared to other 2-Cys PRDXs. This preference is possible due to a more charged amino acid composition in PRDX1, 2 compared to PRDX3, 4. Such composition would be more favourable for a charged compound such as ADNT (see also Supplementary Results).

**Fig. 6:**
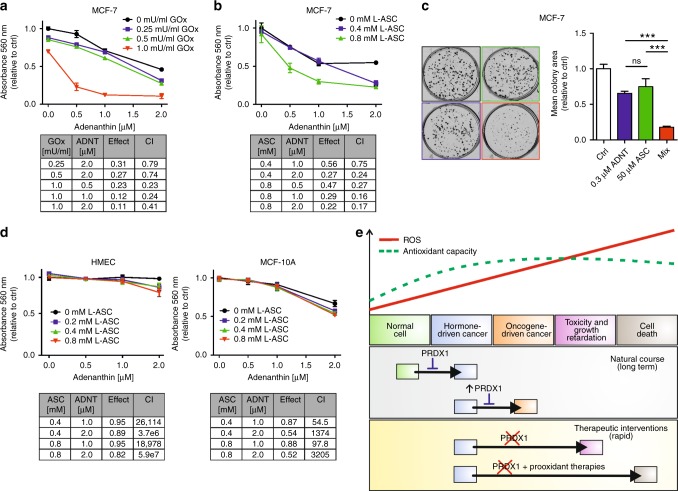
Cytotoxic effects of combinations of adenanthin and GOx or L-ASC in MCF-7 cell lines. Cells were treated with increasing concentrations of ADNT in the absence or presence of either **a** GOx (0.25; 0.5; 1 mU/ml) or **b** L-ASC (0.4, 0.8 mM) for 48 h. At the end of treatment, cell proliferation was determined by crystal violet staining and reported as percent growth relative to controls. The combination index (CI) calculated by the Chou–Talalay method was used to determine drug interaction. The CI is reported at different doses of prooxidants and ADNT as indicated in tables. CI values < 0.9 suggest synergism. **c** Representative results and the quantitative analysis of colony formation assay in MCF-7 cells incubated with 0.3 µM ADNT in combination with 50 µM L-ASC shows a significant decrease in colony area as compared to cells treated with single drugs. Mean ± SEM of the three independent experiments is shown. Statistical analysis was performed with one-way ANOVA followed by Tukey’s honestly significant difference (HSD) post hoc test when significance was detected (***p < 0.001). **d** Non-malignant HMEC (left panel) or MCF-10A (right panel) cells were treated with increasing doses of ADNT in the absence or presence of increasing concentration of L-ASC for 48 h. **e** Scheme representing a dual role for PRDX1 in human breast cancer (please see the text for details)

Herewith, we investigated the effects of pharmacologic inhibition of PRDX1/2 with ADNT in combination with prooxidant agents to test their effects on parental MCF-7 and ZR-75-1 breast cancer cell lines. We have observed that simultaneous treatment of ADNT and GOx resulted in significantly greater growth inhibition than either agent alone in MCF-7 cell lines. The Chou–Talalay combination-index method^15^ using CompuSyn software confirmed the strong synergistic effect observed for both compounds (Fig. 6a). Furthermore, the synergy between ADNT and L-ASC was observed in MCF-7 cell line (Fig. 6b). The same phenomenon was observed when ZR-75-1 cells were treated with these compounds (Suppl. Fig. S8A, B).

The proliferative potential of the MCF-7 or ZR-75-1 cells during treatment with L-ASC in combination with ADNT was also investigated via colony forming assay. As depicted in Fig. 6c (MCF-7) and Suppl. Fig. S8C (ZR-75-1), there was a significant increase in drug-toxicity in combination when compared to single drug treatment. As shown in Fig. 6d, we have observed that HMEC as well as MCF-10A cells were less sensitive to ADNT than MCF-7 cells and insensitive to L-ASC at the concentrations used. Importantly, no positive interaction between the effects of ADNT and L-ASC was seen in both HMEC and MCF-10A cells.

## DISCUSSION

In recent years, there has been a significant progress in the understanding of molecular events contributing to breast cancer. One of the observations was that cancer-related conditions of chronic oxidative stress can have a profound impact on the mammary carcinogenesis process.^35^ However, our knowledge about the specific interplay between prooxidant factors and antioxidant defenses in this disease is far from complete.

The role for PRDX1 in cancer is complex and multifaceted (reviewed in^36^). Importantly for this work, previous publications have indicated that PRDX1 is markedly upregulated in malignant mammary lesions,^16^ as we confirm in our study, which might suggest the tumour-promoting properties of PRDX1. Conversely, *Prdx1*-deficient mice tend to develop, amongst other malignancies, mammary carcinomas,^37^ and, similarly, the previous publication from our group has reported PRDX1 as a favourable prognosis biomarker in ER-positive breast cancer,^9^ which would implicate the role for PRDX1 as a tumour suppressor in mammary malignancies. From these cumulative observations, we conclude that PRDX1 plays in breast cancer a role somewhat similar to oestrogen receptor—i.e., of a “lesser evil”. We propose that although the presence of PRDX1 shields ER-positive breast cancer from progressing into more malignant (i.e. ER-independent) form, at the same time it protects the malignant cells from immediate death and promotes cancerous growth. This suggests that an abrupt inhibition of PRDX1 in breast cancer could be a potential therapeutic modality in this disease, especially when combined with prooxidant therapies. We successfully validated this hypothesis in the current project (graphically summarised in Fig. 6e).

Generally, considering PRDX1 as a therapeutic target in cancer is not entirely a new idea. There are instances, including our own studies, of both genetic and chemical targeting of PRDX1 in such malignancies as oral cancer,^38^ Burkitt lymphoma^8^ or cervical cancer.^39^ Combination of PRDX1 targeting and a prooxidant agent menadione, that induces mostly superoxide production, has also been reported in the past.^24^ However, the significant advantage of the current study is that it pinpoints the unique role for PRDX1, in comparison with PRDX2, as a superior enzyme governing the redox resistance against the exogenous oxidative offence in breast cancer. Notably, we have utilised a genome-editing approach to knockout PRDX1/2 in human cancer cells, which in future can become a clinically applicable modality.

The question shall be addressed how PRDX1 can be specifically targeted in breast cancer cells in a living organism. One of the theoretical possibilities of tumour-specific downregulation of PRDX1 is the conjugation of anti-PRDX1 siRNA with a tumour-specific targeting agent, e.g., an antibody (reviewed in^40^). Targeting CD71,^41^ acting as a transferrin receptor, would be of special interest, as CD71-overexpressing cancer cells are characterised with an increased iron uptake and, hence, should be more prone to prooxidant actions of ascorbate.^42^ Obviously, in HER2-expressing cancers, HER2 molecule itself might be targeted by the siRNA-antibody conjugate. This issue warrants further investigations.

To conclude, in the current study we have shown that PRDX1 is an essential enzyme in managing the H_2_O_2_-mediated oxidative stress in breast cancer cells in vitro and in vivo. Identifying PRDX1 as a dominant antioxidant enzyme in breast cancer cells can have significant implications for understanding the biology and pathophysiology of this disease and future design of prooxidant therapies in mammary malignancies. Indeed, our study highlights potential benefits of combining PRDX1-targeted approaches with agents inducing exogenous production of H_2_O_2_, which gave a dramatic amplification in the efficacy of L-ASC in all malignant cell lines studied. This information is vital, as experiments have shown ascorbate to be effective in killing breast cancer cells for decades,^43^ and yet its effectiveness in clinical trials remains questionable, at best.^44^ However, there is a continued interest in application of pharmacologic ascorbate in anticancer therapies, as exemplified by the recent work by Shoenfeld et al., demonstrating in preclinical and clinical settings the feasibility, selective toxicity, tolerability, and potential efficacy of pharmacological ascorbate in treatment of non-small-cell lung cancer and glioblastoma.^42^ Our present data strongly suggest that the anticancer efficacy of a pharmacological ascorbate might be significantly empowered by combining this compound with targeted suppression of PRDX1 activity in the tumour cells.

### Availability of data and material

The datasets used and/or analysed during the current study are available from the corresponding author on reasonable request.

## Electronic supplementary material

Supplementary Methods

Supplementary Results

Supplementary Tables

Supplementary Figure Legends

Supplementary Figure 1

Supplementary Figure 2

Supplementary Figure 3

Supplementary Figure 4

Supplementary Figure 5

Supplementary Figure 6

Supplementary Figure 7

Supplementary Figure 8

## References

[1] Barrera G (2012). Oxidative stress and lipid peroxidation products in cancer progression and therapy. ISRN Oncol..

[2] Mahalingaiah PK, Ponnusamy L, Singh KP (2015). Chronic oxidative stress leads to malignant transformation along with acquisition of stem cell characteristics, and epithelial to mesenchymal transition in human renal epithelial cells. J. Cell Physiol..

[3] Gorrini C, Harris IS, Mak TW (2013). Modulation of oxidative stress as an anticancer strategy. Nat. Rev. Drug Discov..

[4] Graczyk-Jarzynka A (2017). New insights into redox homeostasis as a therapeutic target in B-cell malignancies. Curr. Opin. Hematol..

[5] Moses C, Garcia-Bloj B, Harvey AR, Blancafort P (2018). Hallmarks of cancer: The CRISPR generation. Eur. J. Cancer.

[6] Rhee SG, Kil IS (2017). Multiple functions and regulation of mammalian peroxiredoxins. Annu Rev. Biochem.

[7] Muchowicz A (2015). SK053 triggers tumor cells apoptosis by oxidative stress-mediated endoplasmic reticulum stress. Biochem Pharmacol..

[8] Trzeciecka A (2016). Dimeric peroxiredoxins are druggable targets in human Burkitt lymphoma. Oncotarget.

[9] O’Leary PC (2014). Peroxiredoxin-1 protects estrogen receptor alpha from oxidative stress-induced suppression and is a protein biomarker of favorable prognosis in breast cancer. Breast Cancer Res.

[10] The Cancer Genome Atlas - Cancer Genome - TCGA http://cancergenome.nih.gov/ Accessed 2017

[11] Robinson MD, McCarthy DJ, Smyth GK (2010). edgeR: a Bioconductor package for differential expression analysis of digital gene expression data. Bioinformatics.

[12] McCarthy DJ, Chen Y, Smyth GK (2012). Differential expression analysis of multifactor RNA-Seq experiments with respect to biological variation. Nucleic Acids Res.

[13] Sanjana NE, Shalem O, Zhang F (2014). Improved vectors and genome-wide libraries for CRISPR screening. Nat. Methods.

[14] Bilan DS (2013). HyPer-3: a genetically encoded H(2)O(2) probe with improved performance for ratiometric and fluorescence lifetime imaging. ACS Chem. Biol..

[15] Chou TC (2010). Drug combination studies and their synergy quantification using the Chou–Talalay method. Cancer Res.

[16] Noh DY (2001). Overexpression of peroxiredoxin in human breast cancer. Anticancer Res.

[17] Zhang D (2016). Reactive oxygen species formation and bystander effects in gradient irradiation on human breast cancer cells. Oncotarget.

[18] Chen WC (2002). Induction of radioprotective peroxiredoxin-I by ionizing irradiation. J. Neurosci. Res.

[19] Jezierska-Drutel A, Rosenzweig SA, Neumann CA (2013). Role of oxidative stress and the microenvironment in breast cancer development and progression. Adv. Cancer Res.

[20] Dickinson BC, Huynh C, Chang CJ (2010). A palette of fluorescent probes with varying emission colors for imaging hydrogen peroxide signaling in living cells. J. Am. Chem. Soc..

[21] Lippert AR, Van de Bittner GC, Chang CJ (2011). Boronate oxidation as a bioorthogonal reaction approach for studying the chemistry of hydrogen peroxide in living systems. Acc. Chem. Res.

[22] Veal EA, Underwood ZE, Tomalin LE, Morgan BA, Pillay CS (2018). Hyperoxidation of peroxiredoxins: gain or loss of function?. Antioxid. Redox Signal.

[23] Carvalho LAC (2017). Urate hydroperoxide oxidizes human peroxiredoxin 1 and peroxiredoxin 2. J. Biol. Chem..

[24] He T, Hatem E, Vernis L, Lei M, Huang ME (2015). PRX1 knockdown potentiates vitamin K3 toxicity in cancer cells: a potential new therapeutic perspective for an old drug. J. Exp. Clin. Cancer Res.

[25] Kim JH (2018). Peroxiredoxin 2 mediates insulin sensitivity of skeletal muscles through regulation of protein tyrosine phosphatase oxidation. Int J. Biochem Cell Biol..

[26] Chen Q, Polireddy K, Chen P, Dong R (2015). The unpaved journey of vitamin C in cancer treatment. Can. J. Physiol. Pharmacol..

[27] Chen Q (2007). Ascorbate in pharmacologic concentrations selectively generates ascorbate radical and hydrogen peroxide in extracellular fluid in vivo. Proc. Natl Acad. Sci. USA.

[28] Niles AL, Moravec RA, Riss TL (2009). In vitro viability and cytotoxicity testing and same-well multi-parametric combinations for high throughput screening. Curr. Chem. Genom..

[29] Park YH (2017). Peroxiredoxin I participates in the protection of reactive oxygen species-mediated cellular senescence. BMB Rep..

[30] Tait L, Soule HD, Russo J (1990). Ultrastructural and immunocytochemical characterization of an immortalized human breast epithelial cell line, MCF-10. Cancer Res..

[31] Lee W (2007). Human peroxiredoxin 1 and 2 are not duplicate proteins: the unique presence of CYS83 in Prx1 underscores the structural and functional differences between Prx1 and Prx2. J. Biol. Chem..

[32] Jiang B (2002). Diterpenoids from Isodon adenantha. J. Nat. Prod..

[33] Muchowicz A (2014). Adenanthin targets proteins involved in the regulation of disulphide bonds. Biochem Pharmacol..

[34] Siernicka M (2015). Adenanthin, a new inhibitor of thiol-dependent antioxidant enzymes, impairs the effector functions of human natural killer cells. Immunology.

[35] Mahalingaiah PK, Singh KP (2014). Chronic oxidative stress increases growth and tumorigenic potential of MCF-7 breast cancer cells. PLoS ONE.

[36] Hampton MB, Vick KA, Skoko J, Neumann CA (2018). Peroxiredoxin involvement in the initiation and progression of human cancer. Antioxid. Redox Signal.

[37] Neumann CA (2003). Essential role for the peroxiredoxin Prdx1 in erythrocyte antioxidant defence and tumour suppression. Nature.

[38] Niu W (2016). Peroxiredoxin 1 promotes invasion and migration by regulating epithelial-to-mesenchymal transition during oral carcinogenesis. Oncotarget.

[39] He T, Hatem E, Vernis L, Huang ME (2014). Peroxiredoxin 1 knockdown sensitizes cancer cells to reactive oxygen species-generating drugs - an alternative approach for chemotherapy. Free Radic. Biol. Med.

[40] Karim ME, Tha KK, Othman I, Borhan Uddin M, Chowdhury EH (2018). Therapeutic potency of nanoformulations of siRNAs and shRNAs in animal models of cancers. Pharmaceutics.

[41] Cinci M, Mamidi S, Li W, Fehring V, Kirschfink M (2015). Targeted delivery of siRNA using transferrin-coupled lipoplexes specifically sensitizes CD71 high expressing malignant cells to antibody-mediated complement attack. Target Oncol..

[42] Schoenfeld JD (2017). O2- and H2O2-mediated disruption of Fe metabolism causes the differential susceptibility of NSCLC and GBM cancer cells to pharmacological ascorbate. Cancer Cell.

[43] Levine M, Espey MG, Chen Q (2009). Losing and finding a way at C: new promise for pharmacologic ascorbate in cancer treatment. Free Radic. Biol. Med.

[44] Jacobs C, Hutton B, Ng T, Shorr R, Clemons M (2015). Is there a role for oral or intravenous ascorbate (vitamin C) in treating patients with cancer? A systematic review. Oncologist.

