# Supplementary material for: Targeting peroxiredoxin 1 impairs growth of breast cancer cells and potently sensitises these cells to prooxidant agents
Source: Br J Cancer. 2018 Oct 5;119(7):873–84. doi: 10.1038/s41416-018-0263-y (PMC6189216; doi:10.1038/s41416-018-0263-y)
Supplement: Supplementary file 1 — Supplementary Methods [file 41416_2018_263_MOESM1_ESM.docx]

**SUPPLEMENTARY METHODS**

**Cell line culture**

All cell lines were maintained through continuous passaging, and were confirmed to be free of contamination by Mycoplasma spp. Cells were cultured with DMEM (ZR-75-1, T47D) or RPMI-1640 (MCF-7, MDA-MB-231, SK-BR-3 and HCC 1806 a kind gift from Dr. Anna Marusiak) media (Sigma Aldrich, St Louis, MO, USA and Gibco by Life technologies, Paisley, Scotland, respectively) supplemented with 10% fetal bovine serum (FBS) (Sigma Aldrich), 2 mM L-glutamine (Sigma Aldrich) and 1% antibiotics – penicillin/streptomycin (Sigma Aldrich) in humidified atmosphere containing 5% carbon dioxide (CO_2_). Additionally, ZR-75-1 and T47D culture media were supplemented with 1nM β-estradiol (Sigma Aldrich). MCF-10A cells, a kind gift from Dr. Anna Marusiak, were cultured in mammary epithelial basal media, (MEBM, Lonza) containing 0.4% bovine pituitary extract (BPE), 10 ng/ml human epidermal growth factor (hEGF), 5 µg/ml human insulin, 0.5 µg/ml hydrocortisone, 30 µg/ml gentamicin and 15 µg/ml amphotericin, and 100 ng/ml cholera toxin (Sigma Aldrich). HMEC cells were cultured in HuMEC medium supplemented with epidermal growth factor, hydrocortisone, isoproterenol, transferrin, and insulin, and 50 µg/ml bovine pituitary extract, according to manufacturer protocol (Life Technologies). The culture media used in this project did not contain sodium pyruvate, as this compound mediates elimination of H_2_O_2_ (Giandomenico *et al ,* 1997).

**RNA Sequencing**

RNA sequencing was performed on amplified fragments of *PRDX1* and *PRDX2* genes covering the designed cleavage site for Cas9. The amplimers were cleaned with Exo/SAP method according to manufacturer protocol (Thermo Fisher Scientific). Then, sequencing reaction was performed for 50 cycles with BigDye Terminator v3.1 Cycle Sequencing Kit and followed by capillary electrophoresis on a 3500xL Genetic Analyzer (Applied Biosystems).

**Western blotting in non-reducing conditions**

Cells were seeded onto 6-well plate at 5 × 10^5^ cells/well in appropriate medium and incubated with increasing concentrations of ADNT (0, 1, 2 μM) for 24 h. At the day of Western blotting procedure, cells were washed twice in PBS and lysed using lysis buffer (50 mM HEPES, pH 7.4, 150 mM NaCl, 5 mM EDTA, 10% glicerol, 1% Triton X-100) supplemented with protease inhibitor cocktail (Roche). Protein concentration was determined using bicinchoninic acid (BCA) method (Pierce, IL, USA). Equal amounts (25 µg) of total protein were loaded on a non-reducing sodium dodecyl sulfate-polyacrylamide gel and resolved by electrophoresis at 90-120 V for 2-2.5 h at 25^◦^C. Separated proteins were transferred onto nitrocellulose membrane. Membrane was then incubated for 1 h at 25°C with 10% nonfat dry milk or 5% BSA in TBS – Tween 20. Afterwards membrane was incubated overnight at 4^◦^C with corresponding antibodies (anti-PRDX1 – 1:1000 dilution, #HPA007730, Sigma Aldrich; anti-PRDX2 1:2000 dilution, #EPR5154, GeneTex; anti-PRDX3 1:1000 dilution, #LF-MA0044, Ab Frontier; anti-PRDX4 1:1000 dilution, #LF-MA0014, Ab Frontier and anti-β-actin-HRP – 1:40000, #A3854, Sigma Aldrich). Blots were exposed to enhanced chemiluminescent substrate (West Femto Maximum Sensitivity Substrate Pierce/Thermo Scientific) and detected using the ChemiDoc Touch imaging system (Bio-Rad Laboratories, Hercules, CA, US).

**Western blotting in reducing conditions**

Cells were seeded onto 6-well plate at 5 × 10^5^ cells/well in appropriate medium. After 24 h, cells were washed twice in PBS and lysed using lysis buffer supplemented with protease inhibitor cocktail (Roche). Protein concentration was determined using bicinchoninic acid (BCA) method (Pierce, IL, USA). Before gel electrophoresis samples were reduced and denatured. Equal amounts (25 µg) of total protein were loaded onto SDS-PAGE. Then, proteins were transferred onto nitrocellulose membrane followed the incubation with 10% nonfat dry milk or 5% BSA in TBS – Tween 20 for 1 h at 25°C. Afterwards membrane was incubated overnight at 4^◦^C with corresponding antibodies (anti-PRDX1; anti-PRDX2; anti-PRDX-SO_3_ 1:250 dilution, #ab16830, Abcam; anti-V5tag 1:5000 dilution, #ab9137, Abcam; anti-phospho-Ser_473_Akt 1:1000 dilution, #4060, Cell signaling; anti-Akt (pan) 1:1000 dilution, #4691, Cell signaling and anti-β-actin-HRP, 1:40000 dilution, #A3854, Sigma). Blots were exposed to enhanced chemiluminescent substrate (West Femto Maximum Sensitivity Substrate Pierce/Thermo Scientific) and detected using the ChemiDoc Touch imaging system (Bio-Rad Laboratories, Hercules, CA, US).

**Redox western blotting**

Cells were seeded onto 6-well plate at 5 × 10^5^ cells/well in appropriate medium. After 24 h, 100 µM H_2_O_2_ was added to medium for 5, 15 and 30 min and incubated at 37 °C . Next, redox western blotting method was applied as described previously (He *et al,* 2015).

Briefly, MCF-7 cells were acid-quenched with ice-cold 10 % TCA, harvested, washed with cold acetone and dissolved in lysis buffer (100 mM Tris-Cl, 1 % SDS, 10 mM EDTA, pH 8.8) containing 25 mM NEM. Following 2 h incubation, under shaking at 30 °C, insoluble protein was removed by centrifugation. Equal amounts (30 µg) of total protein were loaded onto 8-16 % gradient SDS gel (BioRad) under non-reducing condition. Detection of PRDX3 protein on nitrocellulose membrane was performed as described for Western blotting in reducing conditions (see above).

**Irradiation**

Cells undergoing exponential growth were detached from the flask and subjected for irradiation using the prototype Cs-137 device, with an activity of 236 GBq (Giga Becquerel), with 1Gy/h (Grey/hour) dose rate (CLOR, 1987). Samples were exposed to Cs-137 irradiation for 5, 15, and 30 min, which corresponded to doses of 0.08, 0.25, 0.5 Gy, respectively. Control cells were kept in the same experimental conditions but were not exposed to irradiation. Cells were plated in triplicate onto 6‑well plates with a cell density of 1000 cells per well. The cells were then cultured in a 37°C, 5% CO_2_ incubator for 8 days to allow formation of colonies. The colonies were fixed and stained with crystal violet, scanned and counted (see Supplementary Methods).

**Assessment of H_2_O_2_ concentration with PY1 probe**

Cells were seeded onto black, opaque 96-well plates at the density 1.2-1.5× 10^4^ cells per well for 24 h. Next, GOx, at doses of 0.5, 1 mU/ml, was added. Additionally, cells treated with the highest concentration of GOx were preincubated for 30 min with catalase (100 μg/ml), an enzyme specifically removing hydrogen peroxide. Control cells were cultured without any reagent. After another 24 h 10 μM PY1 probe was added to the medium (Sigma Aldrich) for 30 min at 37°C (Dickinson *et al*, 2010; Lippert *et al*, 2011). The read was taken using EnVision reader (Perkin Elmer) at the excitation wavelength 514 nm and emission wavelength 550 nm. The estimated H_2_O_2_ amount was calculated on the basis of non-linear fit to standard curve read-outs prepared with the serial dilutions of 30% H_2_O_2_ solution. After 24 hour incubation with 1 mU/ml glucose oxidase, we detected est. 20 µM H_2_O_2_ concentration in MCF-7 control (parental and sgGFP) cells as well as in sgPRDX2-A/-B MCF-7 clones. In MCF-7 cells with sgPRDX1 knockout, we observed H_2_O_2_ concentration that was approx. three-fold higher – reaching 65 µM.

**Hyperoxidation of PRDXs**

MCF-7 cells were seeded onto 6-well plate at 5 × 10^5^ cells/well in appropriate medium. After 24 h, increasing concentrations of GOx (0, 0.5, 1 mU/ml) were added for next 24 h. Protein lysates were prepared as described for Western blotting under reducing conditions (see Supplementary Methods). The level of hyperoxidation of PRDXs was determined by Western blotting method.

**Generation of MCF-7 sgNTC and sgPRDX1-pool cells for in vivo study**

For production of lentiviral particles, HEK-293T cells were seeded onto 10-cm plates (2.6 × 10^6^ /plate) in DMEM medium and the next day were co-transfected with 8.6 µg of pLenti7.3/V5 TOPO-RedLuc vector, 8.6 µg psPAX2 packaging vector and 5.5 µg pMD2.G envelope vector using calcium phosphate protocol. Briefly, the plasmids were diluted in 450 µl of 250 µM CaCl_2_ and the mixture was added drop by drop to a tube containing 450 µl HBS buffer (0.28 M NaCl, 0.02 M KCl, 0.02 M HEPES buffer, 0.015 M Na_2_HPO_4_, 0.012 M D-glucose). Eighteen hours post transfection, the medium was replaced with fresh DMEM. Supernatants containing lentiviral particles were harvested 48 h after transfection, filtered and concentrated by low-speed overnight centrifugation at 3 000 × *g* at 4°C. The concentrated supernatants were used to infect 0.75 × 10^5^ of target cells. Modified MCF-7-Redluc-GFP cells expressing luciferase and GFP were sorted with FACSAria III cell sorter (BD Biosciences, La Jolla, CA, USA). Then, pools of MCF-7-sgNTC-pool2 and MCF-7-sgPRDX1-pool2 cell lines were generated by CRISPR/Cas9-mediated genome editing, as described in main text.

**MTT assay**

Cells were seeded onto 96-well plates at a density of 1.2-1.5 × 10^4^ per well and treated with appropriate compounds at a final volume of 200 μl. MTT assay was performed as described previously (Trzeciecka *et al*, 2016). Briefly, 25 µl of the tetrazolium dye 3-(4,5-dimethylthiazol-2-yl)-2,5-diphenyltetrazolium bromide (MTT, stock solution 2.5 mg/ml) was added to each well and incubated for 4 h in 37°C. Then culture medium was removed and insoluble formazan was dissolved in 200 µl of DMSO. The absorbance of solution was measured at 570 nm with a plate reader (Asys UVM 340, Biochrom, UK). The optical density reading of five replicates for each treatment was normalized for the mean value of untreated cells and expressed as percentage of control.

**Crystal violet assay**

0.5% crystal violet (Sigma-Aldrich) in 20% methanol was added to each well, and incubated for 15 min at 25^◦^C. The plate was washed in a gentle stream of tap water and air dried. Residual dye was diluted with 2% SDS for 30 min and mixed by orbital shaking at 300-500 rpm. The optical density of each well at 560 nm was measured with a plate reader (Asys UVM 340, Biochrom, UK).

**Propidium iodide flow cytometry-based assay**

Cells were seeded onto 12-well plate at 2.5 × 10^5^ cells/well in RPMI medium. After 24 h, increasing concentrations of either GOx (0, 0.5, 1 mU/ml) or L-ASC (0.2-0.8 mM) were added for next 24 h. Then, cells were trypsinized, washed twice with PBS, resuspended in 200 µl of PBS containing 1µg/ml propidium iodide (PI) and immediately analyzed by flow cytometry. PI fluorescence was determine using the phycoerythrin (PE) channel with a BD FACSCanto™ II Flow Cytometer (BD Biosciences).

**Colony formation assay**

To evaluate the long-term proliferation rate of PRDX1- or PRDX2-downregulated MCF-7 and ZR-75-1 cell lines, modified and control cells were plated in pre-tested appropriate densities yielding 500 into 6-well culture-plates and cultured for 10 days to allow colony formation. To evaluate drug toxicity on the proliferation rate of parental MCF-7 and ZR-75-1 cell lines, cells were plated in densities yielding 1000 into 6-well culture-plates. 24 h after plating, at day 1, cells were incubated with either ADNT or L-ASC alone and in combinations: ADNT:L-ASC for 24 h. Then drug containing medium was removed and cells were cultured in full medium for next 7 days to allow colony formation. After appropriate time, the colonies were stained with 0.5% crystal violet (Sigma-Aldrich) in 20% methanol. Digital images of the colonies were obtained using a BioRad GS-800 Calibrated Densitometer (BioRad), and mean area of colonies were calculated by Fiji software (Schindelin *et al*, 2012) . Experiment was performed in triplicates and repeated three times.

**Viability/cytotoxicity assay**

A total of 1.2 × 10^4^ cells were seeded onto 96-well plate and allowed to adhere overnight. Next, 0.4 mM L-ASC was added, while control cells were incubated only in medium in a final volume of 200 µl. For test, the ApotoxGlo™ Triplex assay kit (Promega) was used. 20 μl of Viability/Cytotoxicity reagent containing both GF-AFC (for viability test) and bis-AAF-R110 (for cytotoxicity test) substrates, was added to each well, and briefly mixed by orbital shaking at 300-500 rpm for 30 seconds and then incubated at 37°C for 60 minutes. Fluorescence was measured at 400_Ex_/505_Em_(viability) and 485_Ex_/520_Em_(cytotoxicity) by using EnVision reader (Perkin Elmer). Experiment was performed in triplicates and repeated three times.

**Cytochemical detection of SA-β-gal activity**

To detect SA-β-gal activity cells were seeded onto 12-well plate at 1 × 10^5^ cells/well in RPMI medium and cultured for 72 h. Next, cells were washed twice with PBS, fixed and stained with the chromogenic β-gal substrate X-gal in a buffer at pH 6.0 according to manufacturer protocol (Cell Signaling). Then, cells were incubated at 37°C for 16 h. After the staining, the cells were washed twice with PBS and viewed by phase contrast microscopy (10× magnification, NIKON).

**Fluorescence detection of SA-β-gal activity**

Cells were seeded onto 12-well plate at 1 × 10^5^ cells/well in RPMI medium and cultured for 72 h. Then, for the next 24 h 0.2 mM L-ASC was added. For measurement of SA-β-gal activity, cells were incubated with a non-fluorescent substrate of SA-β-gal that becomes fluorescent after cleavage by the enzyme (1.5 µl of senescence dye in 500 µl of complete RPMI medium) at 37°C for 2 h. Next, cells were washed twice, harvested by trypsin, and the level of green fluorescent product was detected by flow cytometry using the fluorescein isothiocyanate (FITC) channel with a BD FACSCanto™ II Flow Cytometer (BD Biosciences).

**Fluorescence Activated Cell Sorting**

Cells from 80% confluent culture flasks were detached, centrifuged and resuspended in PBS in final concentration of 5x10^6^ cells/ml. Then, cells were transferred through cell strainer to ensure separation of the cells. The sorting was performed according to intracellular fluorescent protein expression (green fluorescent protein – GFP) with FACS Aria III cell sorter (BD Biosciences, La Jolla, CA, USA).

**Supplementary references**

Dickinson BC, Huynh C, Chang CJ (2010) A palette of fluorescent probes with varying emission colors for imaging hydrogen peroxide signaling in living cells. *J Am Chem Soc* **132**(16)**:** 5906-15

Giandomenico AR, Cerniglia GE, Biaglow JE, Stevens CW, Koch CJ (1997) The importance of sodium pyruvate in assessing damage produced by hydrogen peroxide. *Free Radic Biol Med* 23(3):426-434.

He T, Hatem E, Vernis L, Lei M, Huang ME (2015) PRX1 knockdown potentiates vitamin K3 toxicity in cancer cells: a potential new therapeutic perspective for an old drug. *Journal of Experimental & Clinical Cancer Research* 34:152

Lippert AR, Van de Bittner GC, Chang CJ (2011) Boronate oxidation as a bioorthogonal reaction approach for studying the chemistry of hydrogen peroxide in living systems. *Acc Chem Res* **44**(9)**:** 793-804

Schindelin J, Arganda-Carreras I, Frise E, Kaynig V, Longair M, Pietzsch T, Preibisch S, Rueden C, Saalfeld S, Schmid B et al (2012) Fiji: an open-source platform for biological-image analysis. *Nat Methods*9(7):676-682

Trzeciecka A, Klossowski S, Bajor M, Zagozdzon R, Gaj P, Muchowicz A, Malinowska A, Czerwoniec A, Barankiewicz J, Domagala A, Chlebowska J, Prochorec-Sobieszek M, Winiarska M, Ostaszewski R, Gwizdalska I, Golab J, Nowis D, Firczuk M (2016) Dimeric peroxiredoxins are druggable targets in human Burkitt lymphoma. *Oncotarget* **7**(2)**:** 1717-31
