# Supplementary material for: Targeting peroxiredoxin 1 impairs growth of breast cancer cells and potently sensitises these cells to prooxidant agents
Source: Br J Cancer. 2018 Oct 5;119(7):873–84. doi: 10.1038/s41416-018-0263-y (PMC6189216; doi:10.1038/s41416-018-0263-y)
Supplement: Supplementary file 2 — Supplementary Results [file 41416_2018_263_MOESM2_ESM.docx]

**Supplementary Results:**

**CRISPR/Cas9 introduced alternations on nucleotide level**

RNA sequencing revealed changes in the nucleotide sequence of investigated genes, which resulted in lack of PRDX1 and PRDX2 protein expression.

In case of PRDX1-A (PRDX1-A1, PRDX1-A2 *in navy*) (Suppl. Fig. S1D) both transcripts contain short deletions/omissions. Transcript PRDX1-A1 lacks 15 bp of 5’-end of exon 5, retaining the remaining part of exon 5 and the typical exon 6 translated in correct frame. Transcript PRDX1-A2 lacks the entire exon 5 with the resulting frame-shift for a translation of retained exon 6. The case of PRDX1-B (*in blue*) is identical as the case of transcript PRDX1-A2 described above.

In case of PRDX2-A (PRDX2-A1, PDRX2-A2, *in navy*) (Suppl. Fig. S1E) both of isolated transcripts contain short insertions derived from parts of 5’-UTR of retroviral *gag* gene; their interrelation in *gag* 5’-UTR is shown below (*purple box*). The precise length and site of insertion/insertions of gag 5’UTR to gDNA were not investigated. Additionally, both transcripts lack 15 bp of 5’-end of exon 5 and retain the remaining part of exon 5 and the typical exon 6. In case of PRDX2-A1 the C-terminal part of exon 6 is translated in a correct frame; in case of PRDX2-A2 the transcript is probably much less prominent (or more difficult to amplify), thus the corresponding amplimer was always in minority in relation to PRDX2-A1 and therefore the quality of obtained sequence was insufficient for an unequivocal determination of the reading frame for the retained part of exon 6 (*question mark*). In case of PRDX2-B (PRDX2-B1*, navy-blue*) bases 13 to 183 of exon 6 are missing, resulting in translation of 3’-UTR of PRDX2 instead of its unedited C-terminus.

**Modelling of ADNT interactions with 2-Cys PRDXs**

The active site of 2-Cys PRDXs is composed from two identical subunits (a homodimeric symmetry) which, upon dimer formation, bring together two catalytic cysteines, peroxidatic (Cp) and resolving (Cr) to form a disulfide bond. The structure and sequence comparison of different PRDXs show a highly dynamic, allosteric pocket with many putative conformational changes. For example, the N-terminal loop motif with Cp and the C-terminus where the Cr is located, undergo substantial conformational change during the redox catalytic cycle, and these active site motifs can assume folded or disordered conformation [1].

Among others, adenanthin (ADNT) has been described as an inhibitor of PRDX1 and 2. These two enzymes resemble a high sequence homology (78% identity in their amino acid sequences), with a nearly identical active site. The differences in inhibition can be explained more by the differences in the folded-disordered states of the two enzymes rather than sequence differences [1]. However, other two 2-Cys PRDXs: PRDX3 and 4, have a different active site composition than PRDX1 and 2 (see Suppl. Fig. S7C).

Taking the findings of Liu et al. [2] we have recreated the structure of ADNT-PRDX1 putative binding complex using the humanized rat PRDX1 structure (1QQ2). Next, we manually examined the amino acids composition within 6A from the binding conformation of ADNT in all four PRDX enzymes. The most important differences in case of ADNT binding are Lys120 (PRDX1), Lys119 (PRDX2), Leu176 (PRDX3) and Leu192 (PRDX4) (see Suppl. Fig. S7C, first cyan column). Lysine is a polar, basic amino acid which can form hydrogen bonds with the carboxyl and carbonyl groups of ADNT, as oppose to leucine with non-polar and neutral features. Another important difference between the active sites: glutamic acid with a negatively charged carboxyl group (Glu123 in PRDX1 and Glu122 in PRDX2) is substituted by serine (with an uncharged hydroxyl moiety) in PRDX3 and PRDX4 (see Suppl. Fig. S7C, second cyan column). These changes may further explain differences in binding affinity between PRDX1, 2 and PRDX3, 4. Additionally, Ile125 (PRDX1), Ile124 (PRDX2), Leu181 (PRDX3), and His197 (PRDX4) differ within these proteins. Both, isoleucine and leucine residues are small, nonpolar and hydrophobic, contrary to histidine which is polar amino acid and can be charged (see Suppl. Fig. S7C, third cyan column). This difference could explain poor affinity of ADNT to PRDX4. Finally, a conserved aspartic acid, Asp121 (PRDX1), Asp120 (PRDX2) and Asp193 (PRDX4), is substituted by glycine (Gly177) in PRDX3, which can decrease the number of favorable electrostatic interactions (i.e. hydrogen bonds) in the presence of ADNT (see Suppl. Fig. S7C, single cyan residue). Notably, PRDX5 and PRDX6 differ substantially from the 2-Cys PRDXs in the active site composition. The lack of the required Cys residue on the C-terminus loop makes them unsuitable for ADNT interaction.

In conclusion, the above mentioned differences might explain why ADNT is the most active towards PRDX1 and PRDX2, with less activity against PRDX3 and very minor activity against PRDX4.

**Supplementary references**

1. Wood ZA, Schroder E, Robin Harris J, Poole LB: **Structure, mechanism and regulation of peroxiredoxins**. *Trends Biochem Sci* 2003, **28**(1):32-40.

2. Liu CX, Yin QQ, Zhou HC, Wu YL, Pu JX, Xia L, Liu W, Huang X, Jiang T, Wu MX *et al*: **Adenanthin targets peroxiredoxin I and II to induce differentiation of leukemic cells**. *Nat Chem Biol* 2012, **8**(5):486-493.
