# Supplementary material for: Targeting peroxiredoxin 1 impairs growth of breast cancer cells and potently sensitises these cells to prooxidant agents
Source: Br J Cancer. 2018 Oct 5;119(7):873–84. doi: 10.1038/s41416-018-0263-y (PMC6189216; doi:10.1038/s41416-018-0263-y)
Supplement: Supplementary file 3 — Supplementary Tables [file 41416_2018_263_MOESM3_ESM.docx]

**SUPPLEMENTARY TABLES**

**Table S1.** **sgRNA sequences.** sgRNA sequences were designed using e-crisp website algorithm ([www.e-crisp.org](http://www.e-crisp.org)), only sequences with minimal or none off-target sites were chosen to perform genome-targeted knockout.

| **Name** | **Sequences** |
| --- | --- |
| sgPRDX1 #13 | CCTTATCATCAATGATAAAAAGG |
| sgPRDX1 #14 | ATCAATGATAAAAAGGCCCCTGG |
| sgPRDX2 #12 | GTCTGGTCACGTCAGCAAGCAGG |
| sgPRDX2 #15 | CCACAGTTTGTCCCGCTGGCTGG |
| sgPRDX2 #16 | CTTCCCACAGTTTGTCCCGCTGG |
| sgPRDX2 #44 | GGGAACCCTGATGTCTTCAGGGG |
| sgGFP | GGGCGAGGAGCTGTTCACCG |
| sgNTC | ACGGAGGCTAAGCGTCGCAA |

**Table S2. List of CRISPR/Cas9 modified MCF-7 cell lines generated in the current study**

| **Cell line** | **Description** | **Abbreviated name** |
| --- | --- | --- |
| MCF-7 parental | MCF-7 cell line, original, genetically unmodified | parental |
| MCF-7 CRISPR sgPRDX1#14-pool | MCF-7 cell line carrying a construct with sgRNA (#14) towards PRDX1 | sgPRDX1-pool1 |
| MCF-7 CRISPR sgGFP | MCF-7 cell line carrying a construct with sgRNA towards GFP gene (not present in human genome) | sgGFP-pool1 or sgGFP |
| MCF-7 CRISPR sgPRDX1 clone A | MCF-7 cell line clone A, carrying a construct with sgRNA towards PRDX1 gene | sgPRDX1-A |
| MCF-7 CRISPR sgPRDX1 clone B | MCF-7 cell line clone B, carrying a construct with sgRNA towards PRDX1 gene | sgPRDX1-B |
| MCF-7 CRISPR sgPRDX2 clone A | MCF-7 cell line clone A, carrying a construct with sgRNA towards PRDX2 gene | sgPRDX2-A |
| MCF-7 CRISPR sgPRDX2 cloneB | MCF-7 cell line clone B, carrying a construct with sgRNA towards PRDX2 gene | sgPRDX2-B |
| MCF-7-RedLuc-GFP CRISPR sgNTC - pool | MCF-7-RedLuc-GFP cells carrying a construct with non-targeting sgRNA gene used for in vivo study | sgNTC-pool2 |
| MCF-7-RedLuc-GFP CRISPR sgPRDX1 - pool | MCF-7-RedLuc-GFP cells carrying a construct with sgRNA towards PRDX1 gene used for in vivo study | sgPRDX1-pool2 |
| MCF-7- HyPer-3 CRISPR sgNTC - pool | MCF-7- HyPer-3 cells carrying a construct with non-targeting sgRNA | sgNTC-pool3 |
| MCF-7-HyPer-3 CRISPR sgPRDX1 - pool | MCF-7-HyPer-3 cells carrying a construct with sgRNA towards PRDX1 | sgPRDX1-pool3 |
