# Supplementary material for: Targeting peroxiredoxin 1 impairs growth of breast cancer cells and potently sensitises these cells to prooxidant agents
Source: Br J Cancer. 2018 Oct 5;119(7):873–84. doi: 10.1038/s41416-018-0263-y (PMC6189216; doi:10.1038/s41416-018-0263-y)
Supplement: Supplementary file 4 — Supplementary Figure Legends [file 41416_2018_263_MOESM4_ESM.docx]

**SUPPLEMENTARY FIGURE LEGENDS**

**Figure S1. Characterization of genome-targeted MCF-7 cell lines.** Western blotting for verification of PRDX1 (**A**) and PRDX2 (**C**) protein depletion in MCF-7 cell lines 10 days after transduction, compared to sgGFP control. β-actin was used as a loading control. **B.** A quantitative analysis of colony forming between control (sgGFP) and MCF-7 sgPRDX1-pool1 cells subjected to increasing doses of Cs-137-irradiation (0.08, 0.25 and 0.5 Gy) after 8-day culturing. Control cells were not exposed to irradiation. Experiment was performed in triplicates, and data were obtained from three independent experiments. Statistical analysis was performed with one-way ANOVA followed by Tukey's honestly significant difference (HSD) post hoc test when significance was detected (**p<0.01, ***p<0.001) **D.** Impact of gDNA alterations on PRDX1 transcript level. The schematic diagram of human PRDX1 genomic sequence with exons 1a and 1 to 6 *(black boxes)* is presented under the chromosome 1 ruler (in Mbps) in reverse orientation (*black, on top*), followed by CRISPR target indication *(red triangle)* and a stacked graph of known transcripts from the database of pooled RNAseq experiments showing the relative frequency of splice variants *(green band).* Below RNAseq profile, five known coding transcripts *(navy boxes)* and one noncoding transcript *(light blue)* are schematically presented followed by a graph localizing primer and amplimers for RT-PCR and sequencing *(red arrows)*. The structure of experimentally revealed transcripts is presented *(navy, at the bottom).* **E.** Impact of gDNA alterations on PRDX2 transcript level. The schematic diagram of human PRDX2 genomic sequence with exons 1 to 6 *(black boxes)* is presented under the chromosome 19 ruler (in Mbps) in reverse orientation *(black, on top)*, followed by CRISPR target indication *(red triangle)* and a stacked graph of known transcripts from the database of pooled RNAseq experiments showing the relative frequency of splice variants *(green band).* Below RNAseq profile, six known transcripts are schematically presented *(navy and light blue)* followed by a graph localizing primer and amplimers for RT-PCR and sequencing (*red arrows*). The structure of experimentally revealed transcripts is presented *(navy, at the bottom)*.

**Suppl. Fig. S2.** **Knockdown of PRDX1 inhibits growth of MCF-7 human breast carcinoma in nude mice.** (**A**) Concentration-dependent cytotoxicity of GOx in MCF-7 sgNTC-pool2 and sgPRDX1-pool2 cells. Cells were treated with a GOx (0.25; 0.5; 1 mU/ml) for 24 h. Control cells were cultured without any reagent. At the end of treatment, the crystal violet staining was performed and reported as percent growth relative to control. Statistical analysis was performed with one-way ANOVA followed by Tukey's honestly significant difference (HSD) post hoc test when significance was detected (***p<0.001). (**B**) A single tumor volume plots vs. the number of days post-tumor inoculation of control (sgNTC-pool2) and sgPRDX1-pool2 of MCF-7 cells in mice (initial n=10 mice/group).

**Suppl. Fig. S3.** **Concentration-dependent cytotoxicity of H_2_O_2_ produced by GOx.** (**A**) MCF-7 cells were treated with a range of GOx concentrations (0.5; 1 mU/ml) for 24 h. Cells treated with 1 mU/ml of GOx were preincubated with catalase (100 μg/ml) for 30 min. Control cells were cultured without any reagent. At the end of treatment, the MTT assay was performed and reported as percent growth relative to control. Statistical analysis was performed with one-way ANOVA followed by Tukey's honestly significant difference (HSD) post hoc test when significance was detected (***p<0.001). **B.** MCF-7 cells were treated with increasing concentration of glucose oxidase (0.5 - 1 mU/ml) for 24 h then the level of hyperoxidation of PRDX1-4 was analyzed by Western blotting method. Control cells were cultured without any additional reagent. **C.** Determination of the redox state of PRDX3 in MCF-7 sgGFP and sgPRDX1-deficient cells. Cells were incubated with 100 µM H_2_O_2_ for the indicated time. To analyze the redox state of PRDX3, samples, derivatized with 25 mM NEM, were subjected to SDS-PAGE in non-reducing conditions. β-actin was used as a loading control. Experiment was repeated three times; DIM-dimer, MON-monomer. **D.** ZR-75-1 cells were treated with a range of GOx concentrations (0.5; 1 mU/ml) for 24 h. Experiment was performed as described in Fig. S3A. **E.** Western blotting results showing the phosphorylation status of Ser_473_Akt and total level of Akt in control and genetically modified MCF-7 cell lines. β-actin was used as a loading control. **F.** MCF-7 parental and sgPRDX1-A (*left panel*) and sgGFP, sgPRDX2-A/-B cells (*right panel*) were treated with increasing concentration of glucose oxidase (0.125 - 2 mU/ml) for 24 h, then the phosphorylation status of Ser_473_Akt and total level of Akt was analyzed by Western blotting method. Control cells were cultured without any additional reagent. β-actin was used as a loading control.

**Suppl. Fig. S4.** **Deferoxamine treatment decreases L-ASC-, but not GOx-induced, cytotoxicity in MCF-7 sgPRDX1 cells. A.** Representative examples of light microscopic images of control (parental, sgGFP), sgPRDX1 and sgPRDX2 MCF-7 cells treated without or with L-ASC and DFX alone or in combination for 24 h. Original magnification, ×20. **B.** Control and genetically-engineered MCF-7 cells treated with 0.4 mM L-ASC (**B**) or 0.5 mU/ml GOx (**C**) and 31.25 µM DFX alone or in combination for 48 h. For all cytotoxicity assays, control cells were cultured without any reagent. At the end of treatment, the crystal violet staining was performed and reported as percent growth relative to control (parental). Experiment was performed in triplicates and repeated three times. Statistical analysis was performed with one-way ANOVA followed by Tukey's honestly significant difference (HSD) post hoc test when significance was detected (***p<0.001).

**Suppl. Fig. S5.** **Effect of PRDX1 knockdown on MCF-10A, T47D, MDA-MB-231, HCC1806 and SK-BR-3 cell lines.** Western blotting shows downregulation of PRDX1 protein in MCF-10A (**A**), T47D (**D**), MDA-MB-231 (**G**), HCC 1806 (**J**) or SK-BR-3 (**M**) cell lines after transduction, compared to parental and shNTC controls. β-actin was used as a loading control. Cytotoxic effect of GOx and L-ASC on non-cancerous MCF-10A (**B, C**) and malignant T47D (**E, F**), MDA-MB-231 (**H, I**), HCC 1806 (**K, L**) and SK-BR-3 (**N, O**) cell lines was shown. Cells were treated with increasing concentrations of glucose oxidase (0.0625 – 2mU/ml) and sodium L-ascorbate (0.05 – 1.6 mM) for 24 h. Control cells were cultured without any reagent. At the end of treatment, the crystal violet staining was performed and reported as percent growth relative to control. Experiment was performed in quadruplicates and repeated three times. Statistical analysis was performed with one-way ANOVA followed by Tukey's honestly significant difference (HSD) post hoc test when significance was detected (*p<0.05, **p<0.01, ***p<0.001).

**Suppl. Fig. S6. Effect of PRDX2 knockdown on MDA-MB-231 and SK-BR-3 cell lines.** Western blotting shows downregulation of PRDX2 protein in MDA-MB-231 (**A**), or SK-BR-3 (**D**) cell lines after transduction, compared to parental and shNTC controls. β-actin was used as a loading control. Cytotoxic effect of GOx and L-ASC on MDA-MB-231 (**B, C**), and SK-BR-3 (**E, F**) cell lines was shown. Cells were treated with increasing concentrations of glucose oxidase (0.125 – 2mU/ml) or sodium L-ascorbate (0.1 – 1.6 mM) for 24 h. Control cells were cultured without any reagent. At the end of treatment, the crystal violet staining was performed and reported as percent growth relative to control. Experiment was performed in quadruplicates and repeated two times. Statistical analysis was performed with one-way ANOVA followed by Tukey's honestly significant difference (HSD) post hoc test when significance was detected (*p<0.05, ***p<0.001).

**Suppl. Fig. S7. Adenanthin triggers the disruption of typical 2-Cys PRDXs dimers to enzymatically inactive monomers**. **A.** MCF-7 *(upper panel)* and ZR-75-1 *(lower panel)* cells were cultured with a range of ADNT concentrations (0-2 µM) for 16 h, harvested and analyzed by Western blotting using specific antibodies, as indicated in each blot. Samples were analyzed on the non-reducing denaturating SDS-PAGE. Band migrating at ~22 kDa (labeled MON), corresponds to the molecular weight of the peroxiredoxin monomer, and band migrating at ~ 44 kDa (labeled DIM), corresponds to the molecular weight of the peroxiredoxin homodimer. **B.** Alignment of the amino acid sequences of human 2-Cys PRDXs. Amino acids composing the binding site of ADNT (defined as a 6Å sphere from its putative binding site) are colored. Twelve amino acids (*marked in* *red*) out of seventeen colored are highly conserved. The three main differences in case of physico-chemical features are colored cyan. Columns in yellow highlight additional differences to the binding site that do not directly influence ADNT binding: S126 (referred to PRDX1), the amino acid side chains are directed away from the binding pocket; F127 column is composed of hydrophobic amino acids in PRDX (1-4).

**Suppl. Fig. S8. Cytotoxic effects of combination ADNT with GOx or L-ASC on ZR-75-1 cell lines.** Cells were treated with increasing doses of ADNT in the absence or presence of either **A.** GOx (0.25; 0.5; 1; 1.5 mU/ml) or **B.** L-ASC (0.2, 0.4 mM) for 48 h. At the end of treatment, cell proliferation was determined by crystal violet staining and reported as percent growth relative to control. The combination index (CI) calculated by the Chou-Talalay method was used to determine drug interaction. The CI reported is at different doses of prooxidants and ADNT as indicated in tables. CI values < 0.9 suggest synergism. **C.** Representative results and the quantitative analysis of colony formation assay in ZR-75-1 cells incubated with 0.25 µM ADNT in combination with either 40 µM L-ASC shows a significant decrease in colony area as compared to cells treated with single drugs. Mean ± S.E.M. of the three independent experiments is shown. Statistical analysis was performed with one-way ANOVA followed by Tukey's honestly significant difference (HSD) post hoc test when significance was detected (**p<0.01).
